# Chikungunya Virus Imported into French Polynesia, 2014

**DOI:** 10.3201/eid2010.141060

**Published:** 2014-10

**Authors:** Tu-Xuan Nhan, Aurore Claverie, Claudine Roche, Anita Teissier, Marc Colleuil, Jean-Marie Baudet, Van-Mai Cao-Lormeau, Didier Musso

**Affiliations:** Institut Louis Malardé, Papeete, Tahiti, French Polynesia (T.X. Nhan, A. Claverie, C. Roche, A. Teissier, V.-M. Cao-Lormeau, D. Musso);; general practice, Pirae, Tahiti, French Polynesia (M. Colleuil); Laboratoire Nahoata-Pirea, Pirae (J.-M. Baudet)

**Keywords:** chikungunya, French Polynesia, Pacific, Caribbean, *Aedes*, viruses, mosquitoes

**To the Editor**: Chikungunya virus (CHIKV) is an emerging arthropodborne alphavirus of the family *Togaviridae* (*1*). The most common clinical manifestations of infection with CHIKV are abrupt onset of fever, headache, back pain, myalgia, and arthralgia affecting mainly the extremities; in ≈50% of patients, a rash develops, and relapsing and incapacitating arthralgia is common (*1*). Three CHIKV lineages have been characterized: West African, Asian, and East/Central/South African (*1*,*2*). The strain currently circulating in the Caribbean belongs to the Asian lineage (*2*).

In the Pacific region in 2011, a CHIKV outbreak was reported in New Caledonia (*3*). Additional outbreaks have been reported in Papua New Guinea in June 2012 (*4*), Yap State in August 2013 (*5*), and in the Kingdom of Tonga in April 2014 (*6*). In the Caribbean region, cases of CHIKV infection were reported in the French part of Saint Martin Island in December 2013, after which CHIKV rapidly spread to other Caribbean islands, including Guadeloupe (*2*), where by the end of May 2014 it had caused an estimated 23,100 infections. 

On May 25, 2014, a healthy 60-year-old woman returned to French Polynesia after a 6-month stay with her husband’s family in Guadeloupe, where she had been in contact with family members who reportedly had chikungunya. On the first night after arriving back home in French Polynesia, she noted headache, transient high fever, and mild arthralgia of the knees. The next day, she sought care from her general practitioner for weakness, headache, and severe polyarthralgia (wrists, fingers, knees, toes). Physical examination revealed only swollen fingers and toes; the patient was not febrile. Blood samples were collected, and the patient was administered acetaminophen and corticosteroids. Her headache persisted until day 3, and arthralgia persisted until day 4.

Laboratory tests revealed lymphopenia (589 × 10^6^ cells/L) and slightly elevated C-reactive protein (14.2 mg/L) and liver enzyme levels (aspartate aminotransferase 44 IU/L, gamma-glutamyl transferase 58 IU/L). CHIKV infection was confirmed by a specific real-time reverse transcription PCR (rRT-PCR) with previously reported primers and probe (*7*) and by partial sequencing of the E1 gene (GenBank accession no. KJ939333). Phylogenetic analysis (Figure) showed that the virus strain isolated from the patient was most closely related to strains isolated in the British Virgin Islands in 2014 (VG14/99659, accession no. KJ451624), Yap State, Federated States of Micronesia, in 2013 (FM13/3807, accession no. KJ451622), and Zhejiang Province, China, in 2012 (CN12/chik-sy, accession no. KF318729), with 100%, 99.89%, and 99.78% homology, respectively, thereby confirming its inclusion in the Asian lineage. A blood sample from the patient was inoculated into Vero and *Aedes albopictus* C6/36 cells. Cells were incubated for 6 days, after which time both supernatants were removed and tested. RT-PCR, as described above, gave positive results for CHIKV. 

**Figure:**
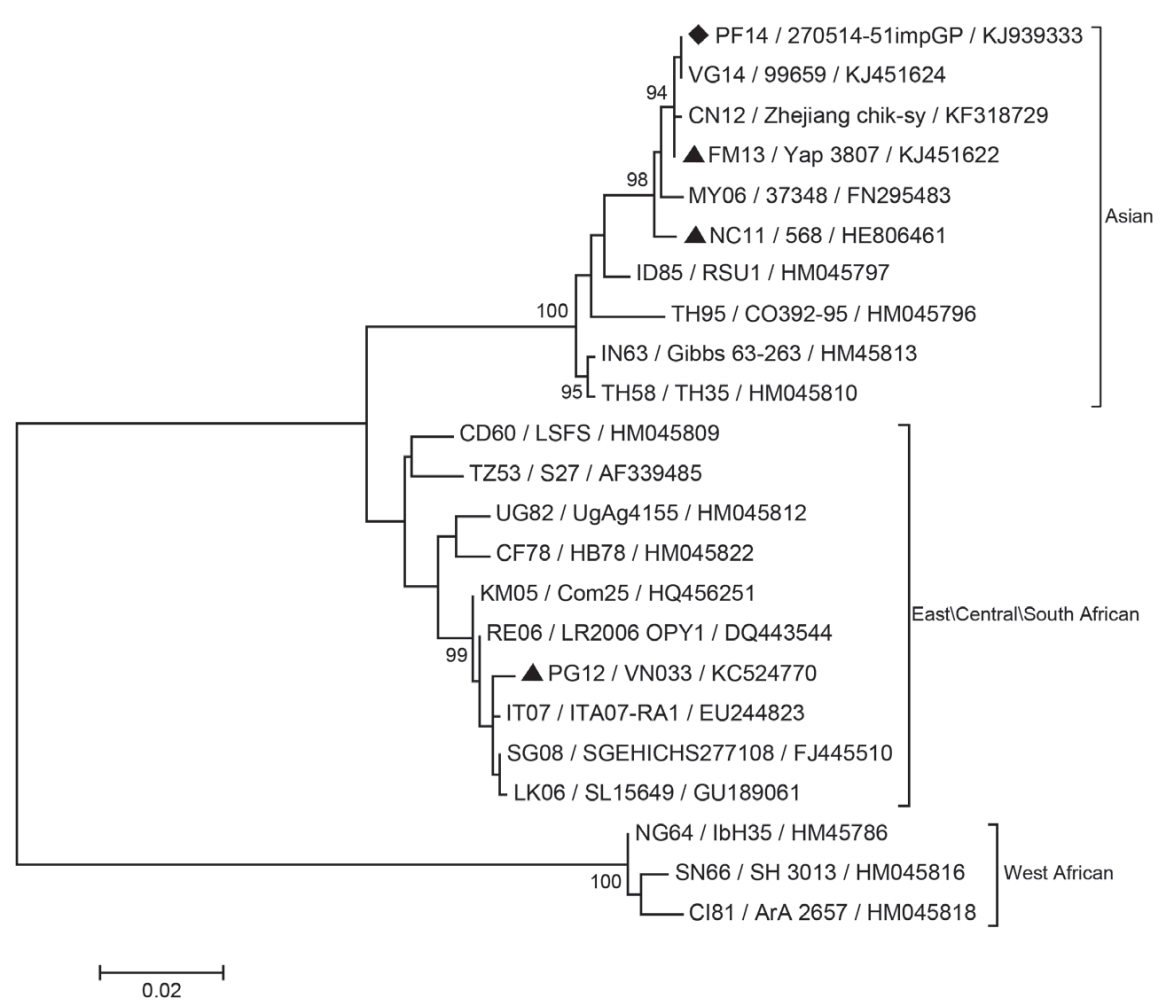
Phylogenetic analysis of the chikungunya virus strain isolated from a patient in French Polynesia, May 2014. The evolutionary history was inferred by using the maximum-likelihood method based on the Kimura 2-parameter model. The percentage of trees in which the associated taxa clustered together is shown for values >90 next to the branches (1,000 replicates). Evolutionary analyses were conducted by using MEGA software, version 6 (http://www.megasoftware.net). Each strain is labeled by country (International Organization for Standardization 2-letter country codes, http://www.iso.org/iso/home/standards/country_codes.htm) and date of origin /strain name/GenBank accession number. Black diamond indicates the chikungunya virus strain from French Polynesia; black triangles indicate strains from other Pacific islands. Scale bar indicates number of substitutions per site.

After CHIKV infection was confirmed, the case was immediately reported to health authorities in French Polynesia. Vector control measures were immediately implemented and included individual protection against mosquito bites (mosquito repellents) for the patient and her close social and family contacts and collective protection (insecticide spraying and breeding site elimination) targeting the house of the patient and the areas that she had visited. Written informed consent was obtained from the patient before publication of this case report.

Arbovirus diseases are endemic to French Polynesia. Dengue virus serotypes 1 and 3 have been co-circulating since 2013 (*8*); and, from October 2013 through April 2014, a large outbreak of Zika virus infection occurred (*9*). Because this case provides evidence of the possible emergence of CHIKV in French Polynesia, health authorities and health care workers in French Polynesia were immediately alerted and prepared to detect local transmission of CHIKV infection.

CHIKV is transmitted by mosquitoes of the *Aedes* species, especially *Ae. aegypti* and *Ae. albopictus* (*1*,*2*). The risk for emergence of a chikungunya outbreak in French Polynesia is high because of the presence of 2 potential vectors: *Ae. aegypti* mosquitoes, vectors of CHIKV in New Caledonia (*3*) and in Guadeloupe (*2*), and *Ae. polynesiensis* mosquitoes, potential CHIKV as suggested by experimental infections (*10*).

The role of foreign travel in spreading arboviruses between French overseas territories is highlighted by the observation that the CHIKV-infected patient reported here returned from Guadeloupe and by a previous report that the 2013 outbreak of dengue virus type 3 in French Polynesia was caused by a virus introduced from French Guiana (*8*). Zika fever was reported in French Polynesia in October 2013; within the next 6 months, 28,000 suspected cases were reported (*9*), but the index patient was not identified. 

As soon as this case of chikungunya in French Polynesia was reported, control measures were applied; as of 4 weeks later, no autochthonous cases have been reported. However, because the French Polynesia population has not been exposed to CHIKV, as it had not been exposed to ZIKV, the possibility of evolution toward a large CHIKV outbreak cannot be excluded. Reinforced epidemiologic surveillance and laboratory capacities are needed for determining whether chikungunya will extend further in French Polynesia and in the Pacific region.
